# Evaluation of a workplace alcohol prevention program targeted on managers’ inclination to initiate early alcohol intervention

**DOI:** 10.3233/WOR-210943

**Published:** 2022-10-17

**Authors:** Devy L. Elling, Ylva B. Almquist, Peter Wennberg, Kristina Sundqvist

**Affiliations:** a Department of Public Health Sciences, Stockholm University, Stockholm, Sweden; b Department of Global Public Health, Karolinska Institutet, Stockholm, Sweden; c Department of Psychology, Stockholm University, Stockholm, Sweden

**Keywords:** Workplace interventions, APMaT, alcohol prevention, hazardous alcohol consumption, inclination to intervene

## Abstract

**BACKGROUND:**

Alcohol interventions targeting the adult population are often conducted in healthcare settings, while preventive interventions often target adolescents or young adults. The general working population is often overlooked. A workplace-based intervention, consisting of development and implementation of an organizational alcohol policy, and skills development training for managers (APMaT) was carried out in order to prevent and reduce alcohol-related harms by identifying hazardous consumers at an early stage.

**OBJECTIVE:**

This study aims to evaluate APMaT by focusing on managers’ inclination to initiate early alcohol intervention.

**METHODS:**

In a cluster randomized design, data were obtained from 187 managers (control: *n* = 70; intervention: *n* = 117). Inclination to initiate early alcohol intervention was measured using three items on a 5-point Likert scale ranging from 1 (strongly disagree) to 5 (strongly agree). Changes in managers’ inclination to intervene were analyzed by applying multilevel ordered logistic regression. Predictors included in the model were group (control vs. intervention), time (baseline vs. 12-month follow-up), and the multiplicative interaction term (group×time).

**RESULTS:**

Significant increase in inclination to intervene against hazardous alcohol consumption among managers in the intervention group compared to managers in the control group was observed. Specifically, a 50% increase of confidence to initiate an intervention was observed among managers in the intervention group.

**CONCLUSIONS:**

APMaT seems effective to increase managers’ inclination to intervene early against hazardous consumption in the workplace. The effectiveness of APMaT at the employee level should be explored in prospective studies.

## 1 Introduction

Hazardous alcohol consumption can negatively affect one’s health (e.g. elevated risk of injuries and cardiovascular- and liver disease) [1], social relationships [2], and the society (e.g. labor market dropouts [3]). Specific to the workplace, employees with hazardous alcohol consumption may miss work (absenteeism) or are unable to perform to their maximum capabilities (presenteeism) [4, 5]. Considering that the majority of the adult population is employed part-time or full-time [6], the costs of absenteeism and presenteeism should incentivize workplaces to prevent hazardous alcohol consumption.

In Sweden, alcohol interventions have traditionally targeted consumers with harmful consumption or dependency in healthcare settings [7], while prevention programs often focus on adolescents or young adults [8, 9]. Consequently, prevention programs [10– 12] conducted in Swedish workplaces are uncommon. The workplace is a favorable arena to identify individuals that may have hazardous alcohol habits [13, 14] since one-third of the adult population spend the majority of their day at work. The workplace also provides an opportunity for a maximum exposure with a stable participation rate to a preventive intervention, and to reach newly employed individuals in the population [15]. Past intervention research have yielded mixed results [11, 16– 20] partly due to the complex interactions of various factors. For instance, one study found that an organizational alcohol policy may be cost-effective in workplaces with limited resources [11], but workplaces with above-average alcohol consumption may benefit more from brief interventions and other forms of alcohol interventions.

In recent years, a guiding framework for effective workplace alcohol prevention programs has been developed, using a whole-of-workplace approach that builds on components such as alcohol policy, work environment, health promotion, and brief interventions [13]. The enforcement of an organizational alcohol policy is influenced not only by organizational attitudes towards alcohol but also by other workplace factors (e.g. alcohol availability, work conditions, and organizational factors) [21], and vice versa. To successfully influence these factors, managers need to improve their routines and understand the complexity of changing workplace practices [22, 23].

The development of a formal alcohol policy occurs at the management level together with human resources (HR) personnel. While implementation of an alcohol policy occurs throughout the organizational levels, managers are often involved in its enforcement. One possible way to ensure the enforcement of the organizational alcohol policy is to incorporate an operational plan, which often includes specific strategies, person responsible, as well as required time frame and resources to enact the strategies [14]. The incorporation of an operational plan in the organizational alcohol policy can be used as a support for managers when they act upon raised concerns regarding their employees’ alcohol consumption [14]. The operational plan can thus facilitate managers to comply with the implemented alcohol policy in the workplace. Therefore, a special focus on managers’ role [22, 23] regarding implementation of a workplace alcohol prevention program should be considered as part of the whole-of-workplace approach.

**Fig. 1:**
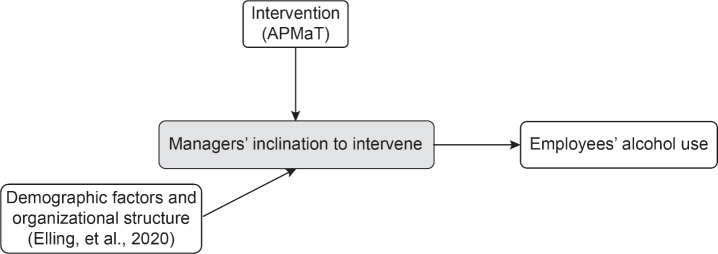
Mechanism of APMaT on managers’ inclination to initiate early alcohol intervention.

The Swedish workplaces are recommended to have an organizational alcohol policy in place [14], although managers often do not have the tools needed for early identification of hazardous consumers [24]. This challenge prompted Alna— a Swedish organization that provides prevention services to workplaces with harmful use of, inter alia, substances [25]— to develop an alcohol prevention program, henceforth referred to as ‘APMaT’ [26]. This program aims to, besides improving alcohol policy, increase managers’ inclination to intervene by raising their awareness of hazardous alcohol consumption and provide information on how to address it. The APMaT program components are hypothesized to improve the identification of hazardous alcohol consumers before adverse effects occur (Fig. 1).

**Fig. 2:**
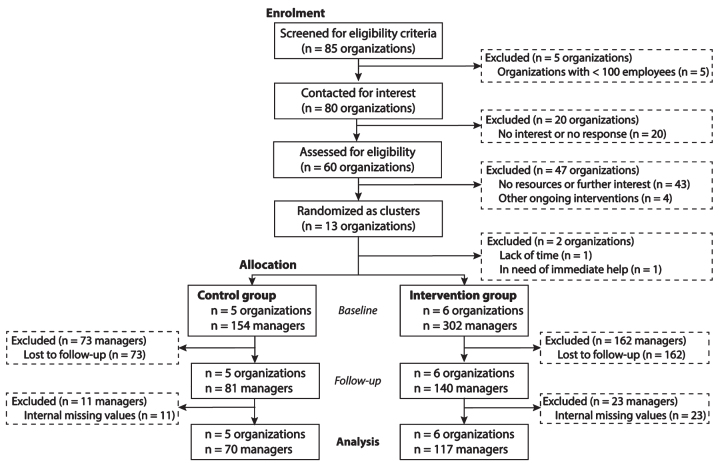
Schematic overview of organizations and participants from recruitment to 12-month follow-up in the cluster randomized controlled study.

The aim of this study is to evaluate whether the APMaT is effective in influencing managers’ inclination to initiate early alcohol intervention. Additionally, the current study seeks to understand the influence of organizational alcohol policy knowledge and action taken upon raised concerns on managers’ inclination to intervene against hazardous alcohol consumption.

## 2 Methods

### 2.1 Study design

This study was based on a larger evaluation project (in English, Controlled study of an alcohol preventive interventions in working life; in Swedish, *Kontrollerad studie av AlkoholPReventiva Insatser i arbetslivet*— KAPRI), that aimed to evaluate the effectiveness of a workplace alcohol prevention program.

The current study was a two-armed, parallel group, cluster randomized controlled study. We chose a cluster design because the prevention program was delivered at the organizational level, and the focus was on workplace policies and managers’ skills.

### 2.2 Recruitment of organizations and participants

KAPRI is a collaborative project between Alna and Stockholm University. Prior to the recruitment process, organizations were first screened through Alna’s company register. Based on the company register, representatives of organizations with at least one hundred employees were contacted via telephone with information on the project’s rationale. Previous research has shown that hazardous alcohol consumption is overrepresented among employees in certain sectors, such as transport, construction, and hospitality [13]. Therefore, organizations in these sectors were prioritized. During the recruitment process, 56 organizations expressed interest and 13 organizations agreed to participate in the project.

Information about the project, including the fact that starting the survey implied the participants’ consent was presented at the start of the survey. Participants were considered eligible for the study if their job description included responsibility for employees. This group thus included supervisors, team leaders, and HR personnel.

### 2.3 Data collection

Data were gathered at baseline (August– October 2018) and at the 12-month follow-up (August– October 2019) through an online survey. The survey was designed to examine managers’ knowledge, attitudes, and practices regarding alcohol in the workplace. Knowledge was measured in terms of knowledge of the organizational alcohol policy, attitudes were measured by inclination to initiate early alcohol intervention, and practices were measured using action taken upon raised concern about alcohol consumption among employees.

A link to the survey was distributed via e-mail, short message service (SMS), and a general link through the organization’s internal website (in cases where e-mails or phone numbers were unavailable). To increase the response rate, all participants who had not completed the survey were sent three reminders via e-mail and SMS at one-week intervals after the initial survey. Participants who received a general link to the survey from the organization’s website were reminded twice at one-week intervals by the organization’s contact person and through the organization’s internal website.

### 2.4 Intervention

APMaT consists of two main components: i) development and implementation of an alcohol policy, and ii) skills development training. During the first component of the intervention, HR personnel and the management team drafted or improved their organization’s alcohol policy, tailoring it to the organizational values together with Alna to sustain its implementation. Specific to the Swedish workplace context, the management team is responsible to record official documentation, and to act as decision-makers for potential strategies in order to ensure the implementation of the alcohol policy. This component was carried out through 2– hour meetings on three to four occasions, depending on the availability of the organization.

In the second component of the intervention– skills development training– managers (including supervisors, team leaders, etc.) completed a two-part workshop with Alna. Each session lasted for 3.5 hours. The first part of the workshop included a discussion on the implementation the organizational alcohol policy, which included difficulties and possibilities for the organization. The second part of the workshop includes activities that aimed to improve their skills at identifying the early signs of hazardous alcohol consumption, and to increase their understanding of the various ways to address alcohol-related issues before adverse effects occur. A detailed explanation of the intervention components is available in the study protocol [27].

### 2.5 Measures

The primary outcome for the study was changes in managers’ *inclination to initiate early alcohol intervention* between baseline and the 12-month follow-up, based on the following statements: “If an employee does not seem to feel well, and it could be due to alcohol use, I feel confident initiating a dialogue about it” (*confidence to initiate a dialogue about alcohol*), “In order to initiate a dialogue with an employee, I first need to be sure that the person has an issue” (*need for confirmation of alcohol problem*), and “In order for an employee to accept help, they need to first acknowledge that they have a problem” (*need for acknowledgement of alcohol problem*). These were measured using a 5-point Likert scale, ranging from 1 (strongly disagree) to 5 (strongly agree). All three items measured different aspects of inclination to initiate early intervention (Cronbach’s alpha = 0.39), and therefore were measured separately.

As potential explanations for any changes identified in managers’ inclination to initiate early alcohol intervention, the study recorded changes in managers’ *organizational alcohol policy knowledge* and *action taken upon raised concerns about alcohol consumption among employees* between baseline and 12-month follow-up. Organizational alcohol policy knowledge was measured using the question: “How well do you know your organization’s alcohol policy?” on a 5-point scale, ranging from 1 (very poorly) to 5 (very well). Managers’ action taken upon raised concerns about alcohol consumption among employees was measured using the question: “During the past 12 months, have you ever initiated a dialogue with an employee because you thought that the person does not feel well?”. This was measured with three response alternatives (no; yes, but only once; yes, twice or more), which were then dichotomized (no; yes).

The study population was described using the following variables in order to examine differences between the control and intervention groups (at baseline and at the 12-month follow-up, respectively): *sex* (male; female), *age* (16– 24; 25– 34; 35– 44; 45– 54; 55– 64;≥65 years), *educational level* (primary; upper secondary; university), *number of supervised employees* (general administrative management, 1– 10; 11– 20; 21– 50;>50 employees), *years in current position* (≤1; 1– 4;≥5 years), *self-rated health* (SRH, very poor; poor; neither poor nor good; good; very good), and *alcohol use* as measured by the Alcohol Use Disorder Identification Test (AUDIT). Due to small cell counts, some of the variables were re-categorized as follow: age (≤34; 25-44; 45-54;≥55 years), educational level (primary/upper secondary; university) and SRH (less than good; good; very good). In addition, total AUDIT scores were dichotomized into abstention and low-risk use, or hazardous use based on the Swedish recommendation, where hazardous alcohol use was defined as≥6 points for females and≥8 points for males [28].

### 2.6 Sample size

Sample size was determined at the organizational level to consider the clustering effect within each organization. In the larger evaluation project, we expected a total of 10-14 organizations with at least one hundred employees in each organization to be sufficient to detect a small effect size (Cohen’s *d* > 0.20) at 5% significance level. The recruited organizations had approximately 20-175 managers depending on their size (medium-sized or large), of which all them were invited to respond to the survey. At baseline, a total of 749 managers were invited to participate in the survey. We expected a response rate of 50% to be able to detect small effect size at 5% significance level.

### 2.7 Randomization and blinding

The thirteen participating organizations were divided into blocks of two to four organizations based on sector (e.g. transportation) and size (medium-sized or large). The organizations within each block was randomly allocated into either the control or intervention group using an online web service (random.org). This randomization procedure was performed to minimize organizational differences between the control and intervention groups at baseline. For instance, two medium-sized organizations within the transportation sector were grouped together, and they were randomly allocated to either the control or intervention group. The organizations assigned to the control group were put on a waitlist control to continue with their usual practices, and the prevention program was delivered to these organizations after the 12-month follow-up.

Due to the design of the prevention program, it was not possible to blind the organizations nor the managers. Further, it was not possible to blind the authors because of the assessment of the waitlist condition. After randomization, two organizations dropped out because they were not satisfied with their group allocation, resulting in a total of 11 organizations (control: *n* = 5 organizations; intervention: *n* = 6 organizations).

The prevention program was delivered by consultants from Alna to each organization. The consultants had two roles: as advisors during the development and implementation of the organizational alcohol policy, and as health educators during the skills development training. The consultants did not have any roles in the evaluation of the program.

### 2.8 Statistical analysis

In order to describe the study population and examine differences between the control and the intervention groups, the frequency and percentages of managers were calculated and compared using Pearson’s chi-square test. Note that for sex, age, and educational level, only differences at baseline were examined. For the remaining variables, differences between the control and intervention groups were assessed at both baseline and follow-up.

As the outcome measure was based on merely two time points, imputation on missing values was considered problematic. Therefore, all analyses followed a complete case analysis approach. Ordered logistic regression with multilevel modelling was applied to analyze the effect of the prevention program on managers’ inclination to initiate early alcohol intervention, where each statement was used as a separate outcome measure. None of the models violated the proportional odds assumption for ordinal outcomes. The choice of applying multilevel modelling was based on the hierarchical structure of the data (organizational and managerial levels), and the ability to include both fixed and random effects. A multiplicative interaction term of group (control vs. intervention) and time (baseline vs. 12-month follow-up) was created to be able to observe changes in the outcome measure.

First, changes in inclination to initiate early alcohol intervention were estimated using group (control vs. intervention), time (baseline vs. 12-months follow-up), and the multiplicative interaction term as the main predictors (Model 1). Next, this model was further adjusted for organizational alcohol policy knowledge (Model 2). The model was additionally adjusted for managers’ action taken upon raised concerns about alcohol consumption among employees (Model 3). Of the variables included to describe manager characteristics, between-group differences were found only with regard to the number of supervised employees, at both baseline and 12-month follow-up (*p* < 0.05). This variable was therefore incorporated in Model 4. In all adjusted models, the inclusion of covariates reflects changes in those covariates between baseline and the 12-month follow-up. All results were presented as odds ratios (OR) with 95% confidence intervals (95% CI). An alpha level of 0.05 was considered statistically significant.

To check for organizational outliers, Model 1 was re-examined by removing one organization at a time. Moreover, compliance effects were assessed by excluding managers who have responded “yes” in the control group and “no/don’t know” in the intervention group regarding their attendance in the skills development training workshops, and subsequently, Model 1 was re-examined.

All analyses were computed using Stata statistical software v.16 (StataCorp LCC, College Station, TX, USA).

## 3 Results

### 3.1 Study population

The analytical sample consisted of those managers who responded to both the baseline and the follow-up surveys and who did not have any missing responses regarding inclination to initiate early alcohol intervention, organizational alcohol policy knowledge, and action taken due to raised concern about alcohol consumption among employees. This resulted in a total of 187 participants (control group: *n* = 70; intervention group: *n* = 117).

At baseline, the majority of managers in both the control and the intervention groups were male (control: 67%; intervention: 72%) and many of the managers were between 35 and 54 years of age. The managers in the control group primarily had a university education (60%), while the largest educational segment for managers in the intervention group was primary/upper secondary education (52%) (Table 1).

**Table 1:** Sociodemographic characteristics of the analytical sample at baseline (n = 187)

Variable, *n* (%)	Control group	*p*-value	Intervention
	(*n* = 70)		group (*n* = 117)
Sex^a^
Male	47 (67.1)	0.501	84 (71.8)
Female	23 (32.9)		31 (28.2)
Age group^a^
≤34 years	7 (10.0)	0.933	11 (9.4)
35–44 years	27 (38.6)		42 (35.9)
45–54 years	27 (38.6)		45 (38.5)
≥55 years	9 (12.9)		19 (16.2)
Education level^a^
Primary/upper secondary education	28 (40.0)	0.108	61 (52.1)
University education	42 (60.0)		56 (47.9)

^a^Differences between the control and the intervention group were calculated using Pearson’s chi-square test

Table 2 describes the differences between managers in the control and the intervention groups at the two time points. At baseline and the 12-month follow-up, both groups generally reported high to very high confidence to initiate early intervention, need for confirmation, and need for acknowledgement from employees regarding hazardous alcohol consumption. With the exception of the statement regarding the need for acknowledgement of problem at follow-up, for which the intervention group reported higher levels compared to the control group, none of the group differences were statistically significant. Moreover, a majority of managers in both groups reported high to very high organizational alcohol policy knowledge. Around one fifth to one fourth of managers in both groups had acted upon raised concerns about alcohol consumption among employees. While managers in the intervention group supervised statistically significantly fewer employees, at baseline and follow-up alike, many of them had been in their current position for longer at baseline. Managers in the control and intervention group had similar SRH and occurrence of hazardous alcohol habits.

**Table 2:** Descriptive statistics of the analytical sample (n = 187)

Variable, *n* (%)	Baseline			12-month follow-up		
	Control	*p*-value	Intervention	Control	*p*-value	Intervention
	group (*n* = 70)		group (*n* = 117)	group (*n* = 70)		group (*n* = 117)
Inclination to initiate early alcohol intervention						
Confidence to initiate early alcohol intervention						
Very low	0 (0.0)	0.368	1 (0.9)	0 (0.0)	0.230	0 (0.0)
Low	5 (7.1)		15 (12.8)	6 (8.6)		19 (16.2)
Medium	13 (18.6)		28 (24.0)	13 (18.6)		26 (22.2)
High	30 (42.9)		48 (41.0)	32 (45.7)		38 (32.5)
Very high	22 (31.4)		25 (21.4)	19 (27.1)		34 (29.1)
Confirmation of alcohol problem						
Very low	7 (10.0)	0.127	11 (9.4)	7 (10.0)	0.666	12 (10.3)
Low	16 (22.9)		15 (12.8)	15 (21.4)		17 (14.5)
Medium	10 (14.3)		33 (28.2)	14 (20.0)		31 (26.5)
High	23 (32.9)		31 (26.5)	18 (25.7)		34 (29.1)
Very high	14 (20.0)		27 (23.1)	16 (22.9)		23 (19.7)
Employees’ acknowledgement of problem						
Very low	3 (4.3)	0.177	3 (2.6)	9 (12.9)	**0.035**	5 (4.3)
Low	13 (18.6)		9 (7.7)	12 (17.1)		9 (7.7)
Medium	12 (17.1)		18 (15.4)	9 (12.9)		15 (12.8)
High	17 (24.3)		38 (32.5)	15 (21.4)		39 (33.3)
Very high	25 (35.7)		49 (41.9)	25 (35.7)		49 (41.9)
Organizational alcohol policy knowledge						
Very low	5 (7.1)	0.341	4 (3.4)	1 (1.4)	0.718	1 (0.9)
Low	3 (4.3)		6 (5.1)	2 (2.9)		5 (4.3)
Medium	16 (22.9)		17 (14.5)	5 (7.1)		14 (12.0)
High	23 (32.9)		38 (32.5)	26 (37.1)		35 (29.9)
Very high	23 (32.9)		52 (44.4)	36 (51.4)		62 (53.0)
Action taken upon raised concerns about alcohol consumption among employees						
No	53 (75.7)	0.738	86 (74.1)	56 (80.0)	0.623	90 (76.9)
Yes	17 (24.3)		30 (23.9)	14 (20.0)		27 (23.1)
Number of supervised employees						
General administrative management	3 (4.3)	**0.027**	19 (16.4)	4 (5.7)	**0.000**	21 (18.1)
1-10 employees	21 (30.0)		36 (31.0)	14 (20.0)		35 (30.2)
11-20 employees	18 (25.7)		17 (14.7)	24 (34.3)		16 (13.8)
21-50 employees	8 (11.4)		21 (18.1)	7 (10.0)		24 (20.7)
>50 employees	20 (28.6)		23 (19.8)	21 (30.0)		20 (17.2)
Years in current position						
<1 year	14 (20.0)	**0.032**	9 (7.8)	5 (7.1)	0.229	9 (7.7)
1-4 years	31 (44.3)		52 (44.4)	40 (57.1)		52 (44.4)
≥5 years	25 (35.7)		56 (47.9)	25 (35.7)		56 (47.9)
Self-reported health						
Less than good	13 (18.6)	0.256	15 (12.8)	22 (31.4)	0.946	36 (30.8)
Good	34 (48.6)		71 (60.7)	38 (54.3)		66 (56.4)
Very good	23 (32.9)		31 (26.5)	10 (14.3)		15 (12.8)
Alcohol habit						
Abstention and low-risk habit	59 (84.3)	0.701	101 (86.3)	60 (85.7)	0.721	98 (83.7)
Hazardous habit	11 (15.7)		16 (13.7)	10 (14.3)		19 (16.2)

Differences between the control and the intervention group were calculated using Pearson’s chi-square test.

### 3.2 Effectiveness of the intervention

Table 3 shows changes in inclination to initiate early alcohol intervention between baseline and 12-month follow-up. Managers in the intervention group reported increased confidence in initiating a dialogue when concerns about alcohol consumption arise among their employees [OR: 1.29; 95% CI: 1.25 to 1.33], even when managers are uncertain whether employees consume alcohol hazardously [OR: 0.91; 95% CI: 0.90 to 0.91]. However, managers in the intervention group reported an increased need for the employees to acknowledge the alcohol problem prior to receiving the help they need [OR: 1.20; 95% CI: 1.17 to 1.23]. After adjusting for alcohol policy knowledge, taking actions, and number of supervised employees, inclination to intervene, the overall patterns remained the same.

**Table 3:** Intervention effect on managers’ inclination to initiate early alcohol interventions in the analytical sample (n = 187). Results from multilevel ordered logistic regression

	Model 1^a^	95% CI	Model 2^b^	95% CI	Model 3^c^	95% CI	Model 4^d^	95% CI
Confidence to initiate early alcohol intervention							
Group, control group = ref.	**0.59**	0.55, 0.63	**0.51**	0.50, 0.51	**0.50**	0.50, 0.51	**0.51**	0.50, 0.52
Time, baseline = ref.	**0.87**	0.86, 0.88	**0.65**	0.60, 0.70	**0.69**	0.68, 0.70	**0.68**	0.68, 0.69
Interaction (group×time)	**1.29**	1.25, 1.33	**1.51**	1.47, 1.55	**1.48**	1.46, 1.51	**1.56**	1.53, 1.58
Confirmation of alcohol problem							
Group, control group = ref.	**1.15**	1.14, 1.15	**1.18**	1.14, 1.23	**1.18**	1.14, 1.23	**1.19**	1.16, 1.22
Time, baseline = ref.	0.99	0.98, 1.01	1.06	0.96, 1.17	1.02	0.90, 1.16	1.02	0.88, 1.17
Interaction (group×time)	**0.91**	0.90, 0.91	**0.87**	0.82, 0.93	**0.87**	0.82, 0.93	**0.85**	0.79, 0.91
Employees’ acknowledgement of problem							
Group, control group = ref.	**1.59**	1.43, 1.77	**1.59**	1.38, 1.83	**1.60**	1.40, 1.82	**1.67**	1.30, 2.15
Time, baseline = ref.	**0.83**	0.81, 0.84	**0.83**	0.76, 0.90	**0.81**	0.71, 0.93	**0.81**	0.70, 0.94
Interaction (group×time)	**1.20**	1.17, 1.23	**1.20**	1.15, 1.25	**1.21**	1.14, 1.27	**1.19**	1.12, 1.27

All results are presented as odds ratios (OR). CI: Confidence Interval. Bold font: *p* < 0.05. ^a^Mutually adjusted for group (control vs intervention group), time (baseline vs 12-month follow-up), and the interaction term (group×time). ^b^Mutually adjusted for group (control vs intervention group), time (baseline vs 12-month follow-up), the interaction term (group×time), and alcohol policy knowledge. ^c^Mutually adjusted for group (control vs intervention group), time (baseline vs 12-month follow-up), the interaction term (group×time), alcohol policy knowledge, and actions taken upon raised concerns. ^d^Mutually adjusted for group (control vs intervention group), time (baseline vs 12-month follow-up), the interaction term (group×time), alcohol policy knowledge, actions taken upon raised concerns, and number of supervised employees.

## 4 Discussion

This study evaluated the effectiveness of APMaT, a workplace-based alcohol prevention program, by investigating changes in managers’ inclination to initiate early alcohol intervention.

The findings show that APMaT seems effective to increase managers’ inclination to intervene against hazardous alcohol habits among employees, at least with regard to dimensions of confidence and need for confirmation. Managers in the intervention group reported the following: an increased confidence and not needing any confirmation in order to initiate a dialogue regarding their employees’ alcohol habit, compared to managers in the control group. Moreover, changes in inclination to initiate early alcohol intervention continued to show positive changes also after organizational alcohol policy knowledge and experiences of action taken upon raised concern was taken into account. The current study findings thus support the effectiveness of APMaT on inclination to intervene, which was one of the main objectives of the project [27].

Our findings are in line with previous literature, where managers perceived an increased knowledge and confidence to initiate early alcohol intervention [10, 29], as a result of combining an organizational alcohol policy and health education. Research on alcohol prevention strategies conducted at the organizational level have consistently illustrate the importance of increasing awareness to be able to influence behavior [10, 30, 31]. For instance, one explanation regarding actual practices of initiating a dialogue may be due to the absence of the need to intervene, rather than a decrease in inclination to initiate an intervention at an early stage. This suggests that a positive change of attitudes through an increase in knowledge is an integral part of altering behavior [31].

Confidence among managers to initiate early alcohol intervention appeared to be the strongest predictor across the three aspects of inclination to intervene. Presumably, an increase of inclination to intervene can improve the way a prevention program is implemented. This could partly be explained by willingness to change at the managerial level [32]. Given that the organizational alcohol policy was tailored according to each organization’s culture and values, the emergence of new workplace routines and cultures could have facilitated implementation processes at the organizational level, by allowing managers an opportunity to reassess their behavior according to the new workplace context [33]. The tailored organizational alcohol policy could also positively alter managers’ attitudes in handling hazardous consumption in the long run, by tackling issues at the highest level within the organization [29]. Although the current study was not able to obtain information regarding structural changes within organizations, this was partly accounted for in the analyses by including fixed effects.

Interestingly, managers in the intervention group continued to report an increased need for acknowledgement from employees prior to receiving the help they need. While the need for acknowledgement is not considered to directly influence managers’ inclination to intervene, it may act as a perceived barrier to initiate intervention at an early stage. The second part of the skills development training therefore attempted to minimize this misconstruction by increasing confidence to initiate a dialogue, even when there might only be a suspicion or a limited risk that employees engage in hazardous alcohol consumption. Based on the results, this part of APMaT did not seem to be entirely effective.

### 4.1 Limitations

In the sensitivity analyses, we did not find any organizational outliers with respect to inclination to intervene against hazardous alcohol consumption.

The survey and program design might have influenced the ability to detect effects from the alcohol prevention program on managers’ inclination to initiate early alcohol intervention. Given that the survey was administered online, responses may have been subjected to social desirability bias, since some of the questions may be of a sensitive nature. Another caveat is the fact that the survey measured managers’ self-efficacy regarding organizational alcohol policy knowledge and inclination to initiate early alcohol intervention, introducing the risk of measurement bias. This plausibly reflected merely managers’ own perception on their ability to act when suspicion arises, rather than a reflection of their actual policy knowledge and practices regarding hazardous alcohol consumption. Finally, because the organizations were recruited from sectors where alcohol consumption among employees tend to be overrepresented, and that the organizational alcohol policy is tailored according to each organization’s values and culture, the results may not be generalizable to other working sectors.

## 5 Conclusions

The current findings indicate that APMaT seems effective concerning managers’ inclination to initiate early alcohol intervention, primarily in terms of increasing levels of confidence to initiate a dialogue with employees about their well-being and alcohol use. The current study contributes to the growing body of intervention research, and could possibly be used as a guide to designing a large-scaled workplace-based alcohol prevention program. Prospective studies should examine the effectiveness of APMaT in the long-run, for instance by investigating changes in alcohol habits in the workplace, particularly at the employee level.
